# Post-Infectious Autoimmunity in the Central (CNS) and Peripheral (PNS) Nervous Systems: An African Perspective

**DOI:** 10.3389/fimmu.2022.833548

**Published:** 2022-03-09

**Authors:** Alvin Pumelele Ndondo, Brian Eley, Jo Madeleine Wilmshurst, Angelina Kakooza-Mwesige, Maria Pia Giannoccaro, Hugh J. Willison, Pedro M. Rodríguez Cruz, Jeannine M. Heckmann, Kathleen Bateman, Angela Vincent

**Affiliations:** ^1^ Department of Paediatric Neurology, Red Cross War Memorial Children’s Hospital, University of Cape Town, Cape Town, South Africa; ^2^ Department of Paediatrics and Child Health, Red Cross War Memorial Children’s Hospital, University of Cape Town, Cape Town, South Africa; ^3^ Paediatric Infectious Diseases Unit, Red Cross War Memorial Children’s Hospital, Cape Town, South Africa; ^4^ Department of Paediatric Neurology, Neuroscience Institute, University of Cape Town, Cape Town, South Africa; ^5^ Department of Pediatrics and Child Health, School of Medicine, Makerere University College of Health Sciences, Kampala, Uganda; ^6^ Laboratory of Neuromuscular Pathology and Neuroimmunology, Istituto di Ricovero e Cura a CarattereScientifico (IRCCS) Instiuto delle Scienze Neurologiche di Bologna, UOC Clinica Neurologica Bologna, Bologna, Italy; ^7^ Department of Biomedical and Neuromotor Sciences, University of Bologna, Bologna, Italy; ^8^ Institute of Infection, Immunity and Inflammation (3I), University of Glasgow, Glasgow, United Kingdom; ^9^ Centro Nacional de Analisis Genomico - Centre for Genomic Regulation (CNAG-CRG ), Centre for Genomic Regulation (CRG), The Barcelona Institute of Science and Technology, Barcelona, Spain; ^10^ Department of Neuromuscular Disease, University College London (UCL) Queen Square Institute of Neurology, London, United Kingdom; ^11^ Faculté de Médecine, de Pharmacie et d’Odontologie, Université Cheikh Anta Diop, Dakar, Senegal; ^12^ Neurology Division, Department of Medicine, Groote Schuur Hospital, Cape Town, South Africa; ^13^ The University of Cape Town (UCT) Neurosciences Institute, University of Cape Town, Cape Town, South Africa; ^14^ Nuffield Department of Clinical Neurosciences, University of Oxford, Oxford, United Kingdom

**Keywords:** post-infectious, immunity, autoimmunity, neurological disorders, encephalitis, encephalopathy, Africa, peripheral nervous system

## Abstract

The direct impact and sequelae of infections in children and adults result in significant morbidity and mortality especially when they involve the central (CNS) or peripheral nervous system (PNS). The historical understanding of the pathophysiology has been mostly focused on the direct impact of the various pathogens through neural tissue invasion. However, with the better understanding of neuroimmunology, there is a rapidly growing realization of the contribution of the innate and adaptive host immune responses in the pathogenesis of many CNS and PNS diseases.

The balance between the protective and pathologic sequelae of immunity is fragile and can easily be tipped towards harm for the host. The matter of immune privilege and surveillance of the CNS/PNS compartments and the role of the blood-brain barrier (BBB) and blood nerve barrier (BNB) makes this even more complex. Our understanding of the pathogenesis of many post-infectious manifestations of various microbial agents remains elusive, especially in the diverse African setting. Our exploration and better understanding of the neuroimmunology of some of the infectious diseases that we encounter in the continent will go a long way into helping us to improve their management and therefore lessen the burden.

Africa is diverse and uniquely poised because of the mix of the classic, well described, autoimmune disease entities and the specifically “tropical” conditions. This review explores the current understanding of some of the para- and post-infectious autoimmune manifestations of CNS and PNS diseases in the African context. We highlight the clinical presentations, diagnosis and treatment of these neurological disorders and underscore the knowledge gaps and perspectives for future research using disease models of conditions that we see in the continent, some of which are not uniquely African and, where relevant, include discussion of the proposed mechanisms underlying pathogen-induced autoimmunity. This review covers the following conditions as models and highlight those in which a relationship with COVID-19 infection has been reported: a) Acute Necrotizing Encephalopathy; b) Measles-associated encephalopathies; c) Human Immunodeficiency Virus (HIV) neuroimmune disorders, and particularly the difficulties associated with classical post-infectious autoimmune disorders such as the Guillain-Barré syndrome in the context of HIV and other infections. Finally, we describe NMDA-R encephalitis, which can be post-HSV encephalitis, summarise other antibody-mediated CNS diseases and describe myasthenia gravis as the classic antibody-mediated disease but with special features in Africa.

## 1. Introduction

Africa is a diverse continent, rich with opportunities. It has a predominantly young population demography with varied socioeconomic backgrounds and human potential. Infectious diseases have plagued the continent in the past, continue to do so in the present and will do so into the future. It is a continent that is also bearing the brunt of the resurgence of previous epidemics and pandemics accompanied by new emerging infections (1–3). “Of 25 countries highly exposed to infectious diseases reported by Infectious Disease Vulnerability Index in 2016, 22 were from the African region” (4). Some of these infections could be prevented with more widespread vaccination or treatments.

Measles is a preventable disease that can have devastating neurological sequelae in those that are not vaccinated, with viral persistence resulting in measles inclusion body encephalitis (MIBE) in immunocompromised individuals, or subacute sclerosing encephalitis (SSPE) in those that are infected in infancy (5, 6). According to the World Health Organization (WHO) Africa there are 26 million Africans infected with HIV (2, 4) with limited access to antiretroviral therapy. Even though combined antiretroviral therapy (cART) has reduced mortality and morbidity of acquired immunodeficiency syndrome (AIDS), it is still endemic, and opportunistic infections (OI) and complications associated with long-term HIV infections, including neurological manifestations, have increased (1). Within Africa clinicians are frequently challenged by the layering effect from multiple influences which impact on clinical disease expression and response to interventions. Co-morbid diseases occur: as an example, vertical transmission or even *in-utero* exposure of HIV, followed by infantile infection with measles typically in the setting of an infant born into a poor socioeconomic environment with limited nutrition and stimulation.

The list of infections with devastating neurological consequences includes Influenza virus, Malaria, Ebola virus, other zoonotic viruses, Onchocerciasis with its recently reported Nodding syndrome, Nakalanga syndrome and other neurological sequelae (1, 7). The interplay between these infectious threats and the peculiar challenges faced by many African countries will result in disastrous consequences, with significant morbidity and loss of life. These other challenges include poverty, malnutrition, poor infrastructure, impact of climate change, political conflict and poor health resources and systems (8, 9).

Despite the advances in antimicrobial treatments and prevention through vaccines and other interventions, neuroinfections continue to ravage populations the world over (10–12). New developments in molecular biology, immunology, better understanding of neuroinflammatory responses and advances in neuroimaging have resulted in better insights into the pathophysiology and impact of neuroinflammation. The role of infections as triggers of autoimmunity in both the central and peripheral nervous systems is being unraveled (13–17). To turn the tide of the scourge of infectious diseases requires innovative approaches and research into the investigation and management of these post-infectious autoimmune disorders.

This review explores the current understanding of the post-infectious autoimmune manifestations of CNS and PNS diseases in the African context. We discuss the proposed mechanisms underlying pathogen-induced autoimmunity, highlight the clinical presentation, diagnosis and treatment of these neurological disorders and underscore the knowledge gaps and perspectives for future research using disease models of conditions that we see in the continent. We will cover para- and post-infectious disease models (see **Table 1**), affecting both the central and peripheral nervous systems in children and adults from an African perspective. Autoimmune encephalitis and myasthenia gravis, each representing central and peripheral nervous systems, respectively, will be presented as they are well studied models of autoimmunity in the nervous system. Despite the paucity of data these conditions also exist and are likely underreported in the African and other resource-limited settings. Data will be presented where available. Awareness needs to be raised and research gaps must be addressed.

**Table 1:** General overview of para- and post-infectious autoimmunity in the central and peripheral nervous systems (see text references).

Disorder	Infectious agent(s)/trigger(s)	Mechanism/Hypothesis	Clinical + Laboratory	Management
Acute Necrotizing Encephalopathy	Influenza A/B, parainfluenza, COVID-19	Cytokine “storm” Genetic predisposition (RANBP2 mutations)	Diagnostic criteria for ANE are as follows (Proposed by Mizuguchi et al.) (18): (1) acute encephalopathy preceded by viral febrile disease; rapid deterioration in the level of consciousness, convulsion; (2) increased cerebrospinal (CSF) protein without pleocytosis; (3) neuroradiologic findings for symmetric, multifocal brain lesions involving bilateral thalami, cerebral periventricular white matter, internal capsule, putamen, upper brain stem tegmentum, and cerebellar medulla; (4) elevation of serum aminotransferase level (5) exclusion of other resembling diseases	Early Immunomodulation (Intravenous methylprednisolone). Supportive.
Measles-associated Encephalopathies: - APME/ADEM - MIBE - SSPE	Measles Virus	Acute Post-infectious/Autoimmune – APME/ADEM Viral Persistence in Immunocompromised host – MIBE Viral persistence/mutation in immunocompetent host	Encephalopathy, multifocal neurological signs and symptoms. Multifocal demyelination (asymmetrical) on MRI. Medically refractory seizures with altered mental status and motor deficits. Usually in immunosuppressed HIV positive patients.	Immunomodulation. Corticosteroids, IVIG Supportive management Antiviral - oral isoprinosine +/- intrathecal interferon
HIV Autoimmune neurological disorders	Human Immunodeficeincy Virus	Attrition and dysfunction of the CD4+ T-lymphocytes, resulting in CD4+ T-lymphocytopaenia. Generation of autoreactive CD8+ T-lymphocytes. Alteration in the balance of regulatory T-lymphocytes and T-helper 17 lymphocytes.	Encephalitis/encephalomyelitis Seizures, encephalopathy, motor paralysis, GBS/polyneuropathy	cART + Immunomodulation (Corticosteroids)
Nodding Syndrome, Nakalanga syndrome and Other Epilepsy	Onchocerca volvulus	Immune-mediated/Autoimmune (Leiomodin-1); other	Affects children. Epilepsy/atonic seizures with head “drops” + other seizure types. Cognitive impairment with neurological regression.	Possible immunomodulation. Not clear yet.
Acute Disseminated Encephalomyelitis	Viruses (eg, measles, mumps, coxsackie, influenza, COVID-19, etc.), Mycoplasma pneumoniae,	Autoimmune; Molecular mimicry. Role of myelin oligodendrocyte glycoprotein (MOG) antibodies.	Encephalopathy, multifocal neurological signs and symptoms. Multifocal demyelination (asymmetrical) on MRI.	Immunomodulation. Corticosteroids, IVIG
Guillain-Barre Syndrome	Campylobacter jejuni, mycoplasma pneumonia, Haemophilus influenzae, EBV, CMV, COVID-19, etc.	Autoimmune. Molecular mimicry. Axonal damage or demyelination. Anti-ganglioside antibodies are detected in some cases, notably *C. jejuni-*related.	Acute flaccid paralysis, with symmetrical areflexic weakness, neuropathic pain, autonomic disturbances, bulbo-respiratory weakness.	Immunomodulation. IVIG or plasma exchange (PLEX). Supportive care.

We hope to reach clinicians and scientists working in neurology, including paediatric and adult neurologists, especially the younger African generation. We also aim to inspire new ways of thinking and dealing with the neuroimmune effects of infections given the continental challenges and current state of understanding. The lessons learnt from the past must be used to impart tools and skills to the next generation of neuroscientists and clinicians for dealing with future challenges in the field of neuroinflammation and autoimmunity.

## 2. Para- and Post-Infectious Neurological Disorders

### 2.1 Neurological Complications of Influenza

Influenza is a single-stranded RNA virus and a member of the Orthomyxoviridae family. Influenza A and B are the major circulating viruses in both adults and children. Influenza epidemics are associated with over 3 million cases of severe illness and about 290 000 – 650 000 deaths, annually (19). The involvement of the nervous system contributes up to 30% of the mortality from influenza in children (19). Minor genetic variations (antigenic drift) are the cause of seasonal variation and larger reassortments generate new strains (antigenic shift) which can lead to pandemic infections in populations with no pre-existing immunity. Influenza viruses primarily cause respiratory illness in humans.

Two forms of central nervous system involvement associated with influenza virus in children and adults are influenza-associated encephalopathy (IAE) and acute necrotizing encephalopathy (ANE) or acute necrotizing encephalopathy of childhood (ANEC) (19, 20). Although initial reports tended to consider IAE and ANE/C together, the current understanding is that ANE is specific, with a characteristic clinico-radiological signature. The role of influenza infection and vaccination in triggering Guillain-Barre syndrome has been extensively studied worldwide since the 1976 swine flu vaccination programme in the USA; however, the risk is unreported in vaccination programmes in Africa and likely to be small (21).

#### 2.1.1 Acute Necrotizing Encephalopathy

The first case series describing ANE was published in 1995 (18, 22). It is a rare but serious and rapidly progressive condition affecting the brain and causing acute swelling and damage of areas of the brain bilaterally, especially the thalami, symmetrical white matter areas and brainstem. Although it is known to affect adults and children, it is more commonly reported in previously well children. It is usually preceded by influenza A (occasionally influenza B) virus or other viral infections (e.g., parainfluenza, HHV6) associated with a high fever (19, 22–24). It manifests with rapidly evolving alteration of consciousness or coma, seizures, subsequent abnormal movements, and other focal neurological complications. ANE is rare but serves as a good learning model in the understanding of the likely immunological processes, genetic interplay, and the severe indirect impact that a common viral infection can have on the CNS (1, 24, 25).

**Figure 1:**
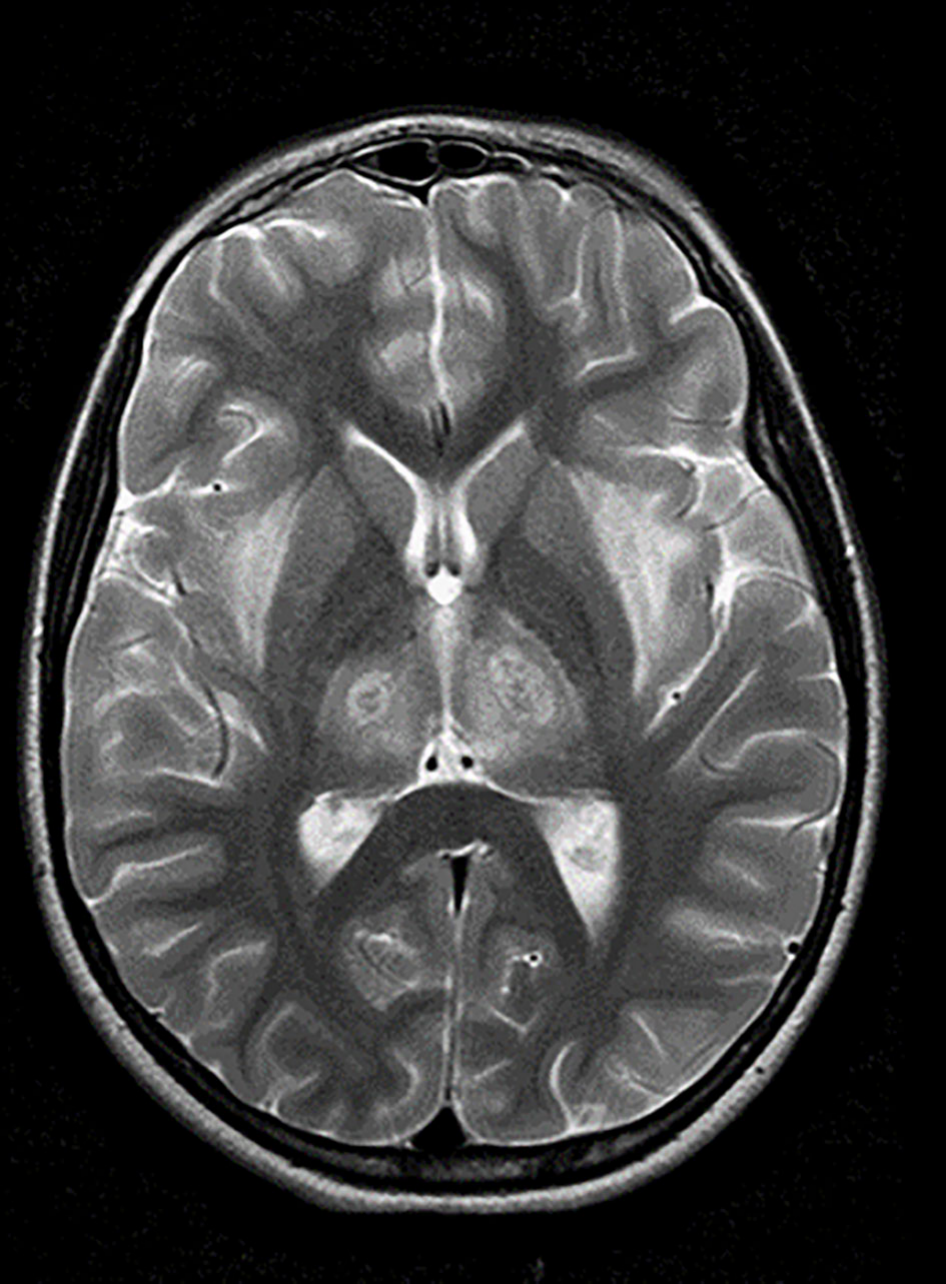
Acute Necrotizing Encephalopathy (Local case). (Personal case of APN and JMW): Axial T-2 weighted MRI of a 9-year-old girl with who presented with classical clinical features of ANE and was admitted to the local paediatric intensive care unit. The MRI shows the classical symmetrical involvement of both thalami (with a target appearance) and symmetrical external capsular white matter affected. She had brainstem involvement (not shown) and was treated with intravenous methylprednisolone early. She survived with mild to moderate neurological sequelae. She was the first in her family to be genetically confirmed as positive for a RANBP2 mutation, with two of her cousins having been previously affected. The genetic result assisted with identification of at-risk family members, counseling and subsequent preventative measures including vaccination and early ANE ‘crisis’ management.

There are two types of ANE, a sporadic type that is not familial and carries minimal risk of recurrence and ANE1 that occurs in genetically predisposed families carrying Ran Binding Protein-2 (*RANBP2*) mutations (24). There may be other genetic factors explaining reports of familial recurrence in individuals without *RANBP2* mutations. Although ANE is prevalent in the Far East and reported in many other parts of the world (mostly Europe and the Americas) (19, 23, 25–28) there is a paucity of reports in the African continent, where influenza and other viral triggers cause much morbidity and mortality. Current authors JMW and APN have anecdotal experience of two extended families that are managed in our centre, from the Western Cape province of South Africa, one with suspected and another with proven *RANBP2* mutations. These families have not been published yet. It is likely, therefore, that more cases are missed or not reported. Despite the lack of resources, molecular and genetic diagnostic tools in many African countries, the clinical-radiological syndrome is quite striking and should enable clinicians with access to magnetic resonance imaging (MRI) on the continent to be able to diagnose the condition (See **Figure 1**). The understanding and exploration of future treatments for this condition, albeit rare, will arm populations vulnerable to influenza epidemics/pandemics in the fight against the neurological consequences that usually follow.

##### 2.1.1.1 Pathophysiology

The clinical manifestations and the classical neuroimaging findings of ANE are well described, with proposed diagnostic criteria (18). However, there has been no evidence of direct infection and neuronal inflammation in the CSF and neuronal tissues. The current hypothesis is one of a cytokine storm due to abnormal nuclear signalling and possible mitochondrial dysfunction (18, 24). Tumour necrosis factor (TNF)-alpha and interleukin (IL)-6 are cytokines that have been consistently shown to be elevated in the serum and cerebrospinal fluid (CSF) (28). Angiopathy, breakdown of the blood-brain barrier and imbalances between the protective and deleterious effects of these cytokines are thought to play a role in the pathogenesis. The CSF usually reveals a raised protein but is acellular. There may be abnormal liver enzymes in the serum. The absence of virus and inflammatory cells in the CSF and neuronal parenchyma indicates lack of direct viral invasion. By contrast, abnormal host responses to viral infection are probably important as different viruses result in a similar clinicopathological picture. The intriguing genetic contribution described in association with *RANBP2* mutations raises opportunities for the clarification of the pathogenesis and ultimately future treatment possibilities (22, 24). *RANBP2* is located on the cytoplasmic surface of the nuclear pore that is a channel that allows small molecules to enter and leave the nucleus by passive diffusion. It is involved in the unpacking, modification and recycling of proteins entering or leaving the nucleus. The exact mechanism by which *RANBP2* mutations result in ANE is not clear but may relate to abnormal mitochondrial interactions (22).

##### 2.1.1.2 Treatment

There are currently no evidence-based treatments for ANE. The place of antiviral (e.g., oseltamivir) treatment is not established as there is no evidence of direct viral CNS infection with this condition. There are anecdotal reports of beneficial effects of immunomodulation, especially intravenous corticosteroids (methylprednisolone) given within the first 24-48 hours of illness, and one retrospective study using high dose intravenous corticosteroids (29, 30). Oseltamivir and/or intravenous immunoglobulins are also added to the corticosteroid regimen by some centers, with variable results (30). Supportive management is crucial, especially intensive care as many patients require ventilatory support. ANE is a fulminant encephalopathy with a variable prognosis. Age, early diagnosis, clinical severity, brainstem involvement on neuroimaging and early treatment with corticosteroids are some of the determinants of outcome in case series. A large proportion of cases reported are left with neurological sequelae with variable but significant mortality of up to 30% (24).

##### 2.1.1.3 Covid-19 (SARS-Cov-2) Associated ANE

The ability of other viruses to trigger ANE suggests a host driven pathologic inflammatory response, and the cytokine storm hypothesis is a common thread linking ANE and some COVID-19-associated diseases. There are a handful of cases recently reported with an ANE-like encephalopathy following Covid-19 (31, 32). There are likely many other cases that have not been reported, and this association needs further study. It is not clear how collision of influenza and COVID-19 would impact the nervous system; it could be a real threat to the continent.

##### 2.1.1.4 Gaps in Knowledge

Beyond anecdotal reports there is a paucity of case reports and research relating to ANE from Africa compounded by limited data on influenza neurological complications. The limited access to laboratory services and neuroimaging (MRI) are related challenges which include lack of resources for sedation/anaesthesia for children (33). Up to now, there is still no clarity regarding the pathogenesis and how *RANBP2* promotes the development of ANE. Early biomarkers for ANE are still under investigation, but CSF neopterin is one of those being looked at (34, 35). Treatment evidence is limited to case reports and case series data. Based on the pathophysiology of the disease, there may be a role for targeted therapies, with inhibition of IL-6 or TNF-alpha, but studies are limited. There are currently not enough data to confirm the association of ANE with SARS-Cov-2. Questions remain regarding the risk of vaccination and immunomodulation treatment implications for influenza associated ANE in people carrying *RANBP2* mutations. The answers will guide the approach to potential SARS-Cov-2 associated ANE.

#### 2.1.2 Neurological Complications of Measles (Wild Type) Virus (MeV)

Measles virus is one of the most contagious diseases in the world. Human beings are the only known natural reservoir (5). It belongs to the Morbillivirus genus of the Paramyxoviridae family and is spread *via* respiratory aerosols. Despite it being vaccine preventable, it continues to be a cause of major morbidity and mortality the world over. There is currently a global resurgence of measles in many parts of the world due to reduction in vaccination coverage, the causes of which differ for various continents (10, 36–38). Reported measles cases increased between 2013 and 2018, with 66% of cases being in low and middle income countries (LMICs) and 23% in persons ≥15 years (10). The global eradication of measles is one of the top priorities of the expanded programme on immunization (EPI), with the support of the World Health Organization (WHO) (37–39). This has been further compounded by the disruption in vaccination roll-out programs during the COVID-pandemic with coverage dropping significantly in some regions (40).

It is a single-stranded RNA virus whose genome encodes six structural proteins. Wild type MeV strains use signalling lymphocytic activation molecule 1 (SLAM or CD150) and nectin-4 receptors to infect target cells (5). The H protein binds to the entry receptor on the host cell surface, whilst the F protein undergoes serial conformational changes, following this attachment. This allows the merge of the host and viral membranes creating a fusion pore to effect viral ribonucleocapsid (RNP) delivery into the host cell cytoplasm (5, 6, 41). Therefore, the H and F proteins constitute the viral fusion complex responsible for viral entry into the host cell. The classic clinical presentation includes, fever, morbilliform rash, oral mucosal Koplik spots, coryza, cough and conjunctivitis. Measles infection can result in several devastating complications, such as pneumonia, immunosuppression, gastroenteritis, and malnutrition.

Neurological complications are less common and include primary measles encephalitis, acute post-infectious measles encephalitis (APME), Measles-inclusion body encephalitis (MIBE) in immunocompromised hosts and Subacute Sclerosing Panencephalitis (SSPE) in those infected at a very young age with or without immunocompromised backgrounds (5). In the South African experience following the 2010 measles outbreak, HIV-exposed or infected children were more predisposed to develop SSPE following MeV infection (42). How the virus enters the CNS is not clear, as the known MeV receptors (SLAM and nectin-4) are not expressed (5, 41). The other complication of MeV infection is the secondary immunosuppression that is induced and can persist for up to 6 months following the initial infection. This immunosuppression will further exacerbate the already negative impact on morbidity and mortality.

##### 2.1.2.1 Acute Post-Infectious Measles Encephalitis (APME/ADEM)

Acute encephalitis occurs within two weeks of initial symptoms and affects about 0.1% of cases. There is no evidence of virus in the brain, and it is thought to be a para- or post-infectious autoimmune disorder similar to acute disseminated encephalomyelitis (ADEM). As in typical ADEM, there is involvement of both white and grey matter with perivenous inflammation and demyelination pathologically (42).

Symptoms include fever, headaches, seizures, focal neurological signs, and encephalopathy. Adults are more likely to suffer from neurological sequelae, and mortality can be as high as 15% (43). Treatment is mostly supportive, but immunomodulation (corticosteroids and intravenous immunoglobulin) has been reported to improve outcomes (43).

##### 2.1.2.2 Measles-Inclusion Body Encephalitis (MIBE)

MIBE occurs in immunosuppressed individuals, usually between 3 weeks and six months following infection with MeV. Unlike APME, MIBE pathology demonstrates evidence of viral entry into the CNS (5, 6, 41). Pathologically, there are intracytoplasmic and intranuclear inclusion bodies (nucleocapsids) in affected neurons, astrocytes, and oligodendrocytes (5, 6). How the virus gains entry without the necessary receptors is a focus of several human and animal studies. Mutations in the HRC domain of the F-protein have been described and are thought to confer an ability for enhanced fusion without the need for H-protein attachment to appropriate receptors (42). These hyperfusogenic MeV mutants demonstrate better viral dissemination without need for H binding (5). Hyperfusogenicity has also been observed in SSPE and therefore MIBE is thought of as a more rapid manifestation of viral persistence in immunocompromised hosts (5, 6).

Clinically, MIBE is a catastrophic form of MeV encephalitis with high mortality and severe neurological morbidity for those that survive. There are medically refractory seizures, altered mental status and associated motor deficits. Up to 75% of those affected succumb following severe seizures and encephalopathy. Status epilepticus is common, often with epilepsia partialis continua (43). In sub-Saharan Africa, this condition has been reported in immunosuppressed patients, mostly adults with HIV infection following MeV epidemics. Neuroimaging is non-specific, may be normal initially and then showing oedema (often along the cortical ribbon) with subsequent cerebral atrophy (44, 45).

##### 2.1.2.3 Subacute Sclerosing Panencephalitis (SSPE)

SSPE affects between 6.5 to 11 cases per 100 000 of immunocompetent patients who contracted the MeV infection in early childhood (5, 43). Almost 100% of those affected will die, usually within 1-3 years of initial SSPE symptoms (5, 46). For those children infected with measles before their first birthday, the incidence can be as high as 1/609 (5). The latency period from infection to symptoms varies from 1 – 15 years. Early signs and symptoms are often non-specific and include mental deterioration, behavioural disturbances, and weakness or impairment of motor function, such as difficulties in walking and frequent falls. Because these features are nonspecific, diagnosis is often delayed, usually years after the initial measles virus infection, especially in resource-limited settings. Later, severe neurologic symptoms such as myoclonic jerks, ataxia, tremors, seizures, and encephalopathy become obvious. The electroencephalogram (EEG) is often abnormal with non-specific slowing initially and followed by the characteristic periodic slow wave complexes (46). Neuroimaging is usually non-specific, ranging from initially normal to cerebral atrophy and white matter hyperintensities later (44, 46). SSPE is characterized by an excessive intrathecal synthesis of MeV specific antibodies in the CSF.

SSPE is almost invariably fatal. In most cases, patients do not survive more than 1–3 years following the appearance of neurologic symptoms. Different drug combinations have been used without much success. There is some anecdotal evidence of longer survival following use of antiviral combination of oral Isoprinosine and intrathecal interferon (5, 46).

##### 2.1.2.4 Gaps

There is still lack of understanding of the factors associated with MeV CNS invasion. It is well understood that an immature immune system before two years of age predisposes to persistent brain infection, but the factors that result in viral persistence are not well known. Viral mutations are thought to play a role in this persistence in the CNS, in both MIBE and SSPE. Regardless of the type of MeV encephalitis, the morbidity and high mortality associated with these complications highlight the need for antiviral treatments against these mutated variants. Vaccination is still the best way to prevent these MeV sequelae, but therapeutic interventions targeting viral entry, CNS dissemination and replication will be crucial for the treatment of CNS infection. International collaborative research into these interventions is urgent in the face of the current global measles resurgence. The coexistence of the HIV epidemic and the global recrudescence of measles in a COVID-19 pandemic melting pot, makes this emergency more acute for Africa.

#### 2.1.3 HIV Infection and Autoimmunity

Southern Africa is the epicentre of the HIV/AIDS pandemic that has ravaged the world since the condition was first recognised clinically in 1981 (47). According to the joint United Nations Programme on HIV/AIDS (UNAIDS), at the end of 2020 the total number of persons living with HIV infection (PLHIV) was 37.6 million, of whom 1.7 million (4.5%) were children less than 15 years of age and 25.3 million (67.3%) were living in sub-Saharan Africa (48).

The clinical progression of HIV infection has been divided into four WHO stages (49). Neurological diseases usually occur in the advanced stages of HIV infection, stages 3 and 4 (50). These diseases can result directly from HIV infection, from opportunistic infections or are thought to be due to autoimmunity. Combination antiretroviral therapy (ART) when administered to HIV-infected individuals will suppress HIV replication, reverse existing HIV-induced immune dysfunction and prevent clinical and immunological progression. However, ART-mediated immunological reconstitution may inadvertently increase the autoimmune risk (51). At the end of 2020, 27.4 million people living with HIV (PLHIV) were accessing ART, a global coverage of 72.9%, and in sub-Saharan Africa 19.5 million people had access to ART, a coverage of 77% (47).

##### 2.1.3.1 Immune Dysfunction

HIV infection causes progressive immunological dysfunction due to the direct effects of HIV on CD4+ T-lymphocytes (CD4 cells), the consequences of virions or specific viral glycoproteins acting on uninfected cells of the immune system, and chronic immune activation arising from the host response to HIV infection (52–54). The rate of progression of HIV infection varies according to age, being more rapid in infants and young children compared to adolescents and adults.

The immunological hallmark in HIV infection is attrition and dysfunction of the CD4+ T-lymphocytes, resulting in CD4+ T-lymphocytopaenia. HIV-induced caspase-3-mediated apoptosis, and caspase-1-mediated pyroptosis triggered by abortive viral infection and chronic immune activation are the mechanisms responsible for most CD4+ T-lymphocyte attrition (55). The immune dysfunction is not confined to CD4+ T-lymphocytes but extends to other components of adaptive and innate immunity, including CD8+ T-lymphocytes, B-lymphocytes, natural killer cells, monocytes and macrophages, neutrophils, and dendritic cells (52, 56–62).

Autoimmunity, caused by a breakdown of immune tolerance and mis-directed immunological responses to self-antigens, can manifest during HIV infection, particularly in the acute stage when the immune system is relatively intact, or after ART initiation during immunological reconstitution when immune competence is restored (51). Several components of the immune dysfunction in HIV infection may contribute to the autoimmunity risk. HIV infection causes polyclonal B-lymphocyte hyperactivation characterised by hypergammaglobulinaemia, increased circulating immune complexes, spontaneously proliferating B-lymphocytes, and production of an array of autoantibodies (63). The release of protein fragments from dying CD4+ T-lymphocytes during HIV infection leads to disruption of tolerance to self-antigens and induces the generation of autoreactive CD8+ T-lymphocytes (64, 65). One study showed that many epitopes on HIV proteins appear to display high similarity with human proteins, suggesting that the induction of cross-reacting immune effectors may be possible (66). Although regulatory T-lymphocytes play important roles in self-tolerance and control of autoimmune diseases, their role in HIV infection remains inconclusive. However, it has been postulated that dysregulation of this cell subset and/or alteration in the balance of regulatory T-lymphocytes and T-helper 17 lymphocytes, may contribute to the breakdown of immune tolerance in PLHIV with autoimmune diseases (67, 68).

##### 2.1.3.2 Autoimmune Neurological Diseases

In a large cross-sectional study of more than 5000 PLHIV the overall prevalence of all autoimmune diseases was 0.7%, but the prevalence of Guillain-Barre syndrome (GBS) was higher in the study population than in the general population (69). In a larger cohort study of more than 33,000 PLHIV, 1,381 (6%) with autoimmune and inflammatory diseases were identified. The only neurological disease reported in this study was multiple sclerosis (70). Similar prevalence studies have not yet been conducted in African countries. Case reports and case series, including studies from Africa have, however, documented central and peripheral nervous system autoimmune diseases in PLHV.

The main autoimmune mediated polyneuropathies in PLHIV are GBS or acute inflammatory demyelinating polyneuropathy (AIDP), and chronic inflammatory demyelinating polyneuropathy (CIDP). GBS usually develops as an ascending polyradiculopathy. In sub-Saharan Africa HIV infection is recognised as an important antecedent infection (71). In PLHIV, GBS frequently occurs in the presence of relatively preserved immunity. However, GBS can be the initial presenting clinical illness in PLHIV or occur after the interruption of ART during viral rebound, and GBS immune reconstitution inflammatory syndrome (IRIS) may present during the first few months of ART (72–78).

Sub-Saharan African studies have described differences in the manifestations of GBS in HIV-infected and HIV-uninfected individuals. In a Zimbabwean study, 16 of 29 patients (55%) with GBS were HIV-infected. Compared to HIV-uninfected patients, the HIV-infected patients with GBS were more likely to have generalised lymphadenopathy, lymphocyte pleocytosis on cerebrospinal fluid (CSF) analysis and co-existent central nervous system (CNS) disease (79). Eleven of 36 GBS cases (31%) from northern Tanzania were HIV-infected. The HIV-infected patients with GBS experienced more severe disease and a higher mortality rate (80). In an Ethiopian study, 19 of 27 GBS patients with HIV serological results were HIV-infected. The clinical findings of the two patient groups were similar, except for a higher frequency of CSF lymphocyte pleocytosis, ventilatory support and mortality among the HIV-infected patients (81).

AIDP and CIDP may be part of a continuous spectrum. However, CIDP differs from AIDP clinically in that CIDP by definition develops over a longer period (greater than 8 weeks) and may follow either a progressive or relapsing remitting course (82). Acute onset CIDP (A-CIDP) may be indistinguishable from AIDP in the early clinical stage. Two South African case series described CIDP in PLHIV. A prospective study described 23 consecutive patients with CIDP during a two-year period, 10 (43%) of whom were HIV-infected. Although not present in all HIV-infected patients with CIDP, CSF lymphocytic pleocytosis was significantly associated with HIV infection. Most of the HIV-infected patients followed a progressive course, while the majority of HIV uninfected experienced a relapsing remitting course (83). The second study was a retrospective comparative review of 84 patients with CIDP, of whom 39 (47%) were HIV-infected. When compared to the HIV-uninfected patients, significantly more HIV-infected patients experienced a progressive course. Median CSF lymphocyte counts were significantly higher in the HIV-infected patients. Most of the HIV-infected patients responded favourably to corticosteroid therapy, and most were in remission by 6 months. These observations suggest that in poor-resourced settings, CIDP in HIV-infected patients should be treated with corticosteroids as the more expensive alternatives such as intravenous immunoglobulin and exchange transfusion are not readily available (84).

Acute disseminated encephalomyelitis (ADEM) a rare demyelinating disorder of the CNS has been documented in HIV-infected children and adults (85, 86). It usually follows a monophasic course. However, multiphasic, or recurrent ADEM, as well as atypical neuroimaging manifestations have been documented in PLHIV (87, 88). Other autoimmune neurological diseases have been documented in PLHIV including myasthenia gravis, N-methyl-D-aspartate-receptor antibody encephalitis, HIV-associated opsoclonus-myoclonus-ataxia syndrome, and neuromyelitis optica with or without detectable anti-aquaporin-4 autoantibodies (89–92). Inflammatory neurological diseases caused by unknown mechanisms have also been documented in PLHIV. One such disease is HIV-associated CD8+ T-lymphocyte encephalitis, a rare inflammatory disease that has not yet been reported from Africa but is characterised by the infiltration of the brain by CD8+ T-lymphocytes in the absence of opportunistic infection. Important risk factors include interruption of ART and IRIS after ART initiation. Although it is not known whether autoimmune mechanisms underpin this disease, treatment with corticosteroids improves outcome by reducing mortality (93, 94).

##### 2.1.3.3 Research Priorities

This review identified major knowledge gaps. Studies that utilise advanced immunology and molecular techniques including whole genome sequencing and transcriptomic profiling are needed to advance the pathogenesis of HIV-associated autoimmune diseases. Addressing diagnostic constraints that exist in Africa, including limited neuroimaging facilities, and pathology and immunology support, is required to improve the recognition of neuro-autoimmune diseases in PLHIV. Optimal disease recognition is a prerequisite for undertaking comprehensive epidemiological studies to understand the incidence, autoimmune spectrum, risk factors and autoimmune risk over the course of ART in African settings. Improved diagnosis should also assist in optimising treatment interventions for these diseases through adequately powered, multi-centre, randomised intervention studies.

#### 2.1.4 Onchocerciasis

##### 2.1.4.1 Disease Description

Onchocerciasis is a neglected tropical parasitic disease with an estimated 20.9 million infected people worldwide, more than 99% of whom reside in 31 countries in sub-Saharan Africa (95). Currently in Africa, 218 million people live in areas known to be endemic for onchocerciasis, a disease induced by infection with the filarial nematode *Onchocerca volvulus* (*O.volvulus*) transmitted by Simulium spp. (blackflies) which inject larval stage 3 (L3) into the skin of the host during a blood meal (96, 97). The larvae eventually develop into adult microfilariae (mf) which localize to the subcutaneous nodules where they may exist for up to 15 years (98). The death of these mf provokes an inflammatory immune response, which is the key feature of the clinical manifestations of onchocerciasis infection observed in the eye, skin, and the nervous system (99).

Eye manifestations include features of chronic keratitis and sclerosis leading to ongoing loss of corneal clarity and peripheral vision as well as corneal fibrosis and or opacification that progresses to blindness (100). In addition, the eye features may be complicated with secondary glaucoma of the anterior and posterior segment lesions and optic atrophy (101).

Skin manifestations include an Onchodermatitis which may be acute causing an itchy skin rash of small, sparse popular lesions or closely packed papules of about 1mm radius, while the chronic form manifests with a pruritic, hyper pigmented, flattopped papulomacular rash of about 3mm with or without skin excoriation (102). The chronic form may result in raised, discrete, pruritic hyperpigmented papular nodules termed as ‘onchocercoma’ which are found around bony prominences such as the iliac crest, ischial tuberosity, elbows, and scapula, If the chronic dermatitis is characterized by hyperpigmented papules and regional lymph nodes enlargement it is referred to as *“Sowda*” (103, 104).

Neurological manifestations include Onchocerciasis-associated epilepsy (OAE). It has been suggested that the clinical presentation of the Nakalanga syndrome and Nodding syndrome form part of the spectrum of OAE (99). The Nakalanga syndrome first described in Uganda in 1950 is characterized by unexplained growth retardation commonly affecting children aged 3-18 years that were previously on the normal growth trajectory (105). Other features include delayed sexual development, intellectual disability, facial, thoracic, and spinal abnormalities with or without epileptic seizures (106–108). Nodding syndrome (NS) is a progressive, epileptic syndrome of undetermined aetiology, affecting previously healthy children with normal growth between the ages of 3 and 18 years (109). Typical features of the syndrome include fleeting episodes of a sudden onset of head nods (atonic seizures) (102). Other seizure types include myoclonic-, absence- and/or generalized tonic–clonic seizures which may commence 1–3 years after the onset of the illness (110). Additional features include deteriorating cognitive and motor function, stunted growth, psychiatric disorders, progressive generalized wasting, and physical deformities (109).

OAE encompasses a large variety of seizures, such as atonic neck seizures (seen in NS), myoclonic neck seizures, absences without nodding, and generalized tonic-clonic seizures. Initially children may manifest with the atonic type of seizures seen in NS and gradually develop generalized tonic-clonic seizures as they advance in age (111). In addition, in some cases impaired cognitive function, malnutrition, dysmorphic features, with arrested sexual debut as seen in Nakalanga syndrome may be associated with OAE (112). A case definition for OAE has been proposed to fulfil at a minimum all the following criteria namely: the age of onset between the ages of 3–18 years old; a history of two unprovoked seizures 24 hours apart; normal psychomotor growth trajectory prior to onset of symptoms; individual from area of high epilepsy prevalence with other siblings affected by epilepsy; and having lived at least three years in an onchocerciasis endemic region (99).

##### 2.1.4.2 Pathophysiology: How the Organisms Induce CNS Disease

The pathophysiological mechanism by which the *O.volvulus* causes disease in the CNS remains poorly understood with no clear consensus on whether or not the mf can cross the blood brain barrier and conflicting reports regarding the presence of *O.volvulus* in the cerebrospinal fluid (113, 114). There is evidence to support two postulated modes of entry which include *via* the eye and the blood stream. Mf have been isolated in the posterior section of the eye suggesting a possible channel of transmission along the inflamed optic nerves which have proximity with the brain (115). Conversely, the presence of microfilariae in the bloodstream and lymphatic system of heavily infected individuals, may cross the blood brain barrier when flowing through the subarachnoid space (116). Recent reports suggest an immune-mediated mechanism rather than direct CNS invasion, as described below.

##### 2.1.4.3 The Interplay Between the Organisms and the Immune System in the CNS, and the Consequences Thereof

There are three proposed mechanisms that illustrate how the *O.volvulus* organisms interact with the immune system in the CNS to cause complications. In the ocular system, there is a cross reaction between the Ov39 antigen of *O.volvulus* and the retinal hr44 antigen which plays a significant role in the development of chorio-retinitis (117). The bacterium *Wolbachia* co-exists with the *O.volvulus* and the other cross reaction also occurs in the ocular system following the stimulation of a Th1-mediated host immune response due to the release of *Wolbachia* surface antigens succeeding the death of the mf. This reaction contributes to the progressive visual impairment seen in Onchocerciasis (118).

More current evidence suggests that OAE, (specifically NS) may be as a result of cross-reacting antibodies between the human protein leiomodin-1(LM1) and the *O. volvulus* surface protein tropomyosin causing an autoimmune reaction (119). LM-1 is a protein present in neurons, muscle tissue and the thyroid gland of healthy individuals and anti-LM1 antibodies were found to be more common in the serum of NS patients compared to controls. In addition, they were also detected in cerebrospinal fluid (CSF) of persons with NS and noted to be neurotoxic *in vitro* (119).

##### 2.1.4.4 Value of Neuroimmune Changes in Diagnostics and Therapeutics

The cell-mediated immune response in the host during early *O. volvulus* infection is markedly increased compared to the chronic infection state where it is diminished for reasons that are still not clear (120). *O. volvulus* infection has been noted to work against the immune responses of the host through molecular mimicry, by impairing T-cell activation and interfering with the processing of antigens (121–123). Furthermore, it has been shown that antigen specific regulatory T-cells (Tr1/Th3) generate anti-inflammatory cytokines, including IL-10 and transforming growth factor-β, which aid in the evasion of host immune responses by O. volvulus. The presence of IL-10 suppresses the Th1-immune response, thereby promoting chronic onchocerciasis (124, 125).

Infection with *O. volvulus* also affects the host’s resistance to other diseases, for example increased probability of becoming HIV-positive when exposed to it compared to non-onchocerciasis individuals or a greater susceptible to developing epilepsy, which may all result in a reduced life expectancy of the host (124, 126–129).

Valuable diagnostic tools for *O. volvulus* infection are available which are efficient for individual use, such as skin snips for demonstrating microfilariae/adult worms in nodules excised or detection of *Ov*-specific antibodies, such as the Ov16 serological test (130). On the other hand, use is made of the diethylcarbamazine (DEC) patch test to evaluate the levels of endemicity and to detect recurrence of transmission in previously controlled areas for community-based onchocerciasis control needs (131). Current infection of *O. volvulus* can also be identified *via* DNA polymerase chain reaction or *O. volvulus* antigens *via* immunoblotting or a dipstick assay (132–134).

The approved therapy for mass treatment of onchocerciasis is the drug ivermectin, which enhances immune responses against *O. volvulus* in the treated host (135). The immune response increases the number of circulating CD4 + T-cells resulting in a significant reduction of microfilariae (136). However, repeated cycles of treatment with this drug are warranted in view of its inability to kill the adult worms (137). Doxycycline, an alternative therapy works by significantly reducing the life span of the adult worm through its action on the endosymbiotic Wolbachia bacteria of *O. volvulus*. Caution however should be observed in the simultaneous use of these drugs since the treatment interactions have not been elucidated.

##### 2.1.4.5 Knowledge Gaps

Important gaps in knowledge include understanding the pathophysiological mechanisms that enable the host with prolonged *O. volvulus* infection to have a blunted cellular immune response compared to those with early infection and what determines the *O. volvulus* parasite to trigger development of Nodding syndrome, Nakalanga syndrome or OAE. Information on a more precise estimation of the burden of OAE globally is needed to guide governments and international onchocerciasis elimination programs to set up relevant interventions. Furthermore, to ascertain whether treatment with Ivermectin and doxycycline can modify the clinical presentation of OAE by decreasing its incidence.

#### 2.1.5 Guillain-Barre Syndrome (GBS)

The acute post-infectious paralytic disorder termed GBS occurs worldwide with an annual incidence of 1-2 cases per 100,000 of population (138). Case series and population studies on GBS have been widely reported throughout North and Sub-Saharan Africa that in general follow the clinical and epidemiological patterns to those seen in other parts of the world with similar environmental factors (139). The age distribution of GBS in Africa tends to be younger than that reported in Europe and North America, most likely a reflection of the general population age. The background infections that trigger GBS are very dependent upon climatic region and environmental factors including epidemic and endemic events and thus likely to have a major influence on the overall pattern and incidence of disease, as seen for example during the Zika virus epidemic (140). The global effort in GBS research has recently accelerated, owing in part to the huge success of the multinational International GBS Outcome Study (IGOS) run from Erasmus University, Rotterdam, that includes input from South Africa (141). A summary of the first 100 years of GBS global research can be found in the freely downloadable book edited for the GBS centenary meeting held in 2016 (142).

##### 2.1.5.1 Differential Diagnosis of GBS

The accurate and confirmatory diagnosis of GBS and its sub-type categorisation is highly dependent upon access to electrodiagnostic testing and CSF examination, procedures whose availability is generally limited to specialist referral centres. Without access to these diagnostic procedures there is considerable diagnostic uncertainly based solely on clinical features, as reflected in levels of certainty described in the widely used Brighton Criteria classification system (143). Since GBS essentially presents as an acute flaccid paralysis in both adults and in children, the extensive differential diagnosis includes a wide range of infectious, inflammatory, metabolic, vascular, and toxic events. Some of these, such as polio and rabies may be highly location- and time-specific; others, such as *Campylobacter jejuni* enteritis and HIV infection (see section 2.3 above) are more widespread (144). In children, where the clinical manifestations may be atypical, the diagnosis of GBS may be particularly difficult to firmly establish. The relationship between SARS-Cov-2 and GBS has yet to be fully clarified in Africa and elsewhere, although cases of GBS have been reported (145). A recent case series reports an increased risk of GBS following SARS-Cov_2 infection and after COVID-19 vaccination. There was a greater risk of complications following SARS-Cov-2 infection compared with the observed vaccination risk (146).

##### 2.1.5.2 Treatment and Management of GBS

The gold standard of current for GBS has recently been summarised in an easy-to-follow 10 steps article taking into account the current evidence-based guidelines (143). This guideline article is currently undergoing translation into languages other than English and includes a simple wallchart in the first figure. In many parts of the world the 2 proven treatments – plasma exchange and intravenous immunoglobulin therapy - are neither available nor affordable and other measures thus need to be considered. Small volume plasma exchange is an alternative approach in resource limited settings that has undergone a recent re-evaluation in Bangladesh (147). Since around 30% of GBS cases require intensive care with mechanical ventilation in order to survive the acute phase of the illness, rapid access to these facilities is required. In addition to specific immunotherapy and intensive therapy support, it is equally important to consider the wide range of early and late complications that arise when managing GBS cases, including aspiration pneumonia and lung injury, cardiac arrhythmia, deep venous thrombosis, limb contractures and pressure sores, and mitigate against these in the management plan. Outcome can also be predicted using a variety of rating scales. The mortality of GBS is clearly predicated upon the level of acute supportive care that can be provided; even in the best clinical settings mortality is around 5%, and 20% of surviving patients have significant long-term residual disability. Long-term clinical monitoring is not usually required for patients who recover well. The recurrence rate is low (<5%). As mentioned above, some patients with A-CIDP may present as GBS and thus will require a different treatment plan.

## 3 Well Established Post-Infectious or Tumour-Related Autoantibody-Mediated Disorders

### 3.1 NMDAR-Antibody Encephalitis

Autoimmune encephalitis (AE) mediated by antibodies against neuronal surface antigens (NSA-Ab) represent an expanding spectrum of immune mediated disorders characterized by the subacute onset of complex neurological and psychiatric symptoms usually responsive to immunotherapy (148). Several antibodies have been identified so far, (as shown in **Table 2**); NMDAR, LGI1, CASPR2 and GABAAR antibodies are the most commonly found, although data from within Africa are very limited. Here, we will focus on NMDAR-antibody encephalitis, since it is the only one for which an infectious trigger has been recognised, at least in some cases.

**Table 2:** Clinical features of main forms of autoimmune encephalitis (AE) associated with known specific antibodies against neuronal surface antigens.

Antigen	Median age (range)	Sex ratio (M:F)	Main clinical syndrome	Other syndromes	Imaging	CSF features	Other features	Associations	Outcome
**Antigens with well-known neuronal roles – excitatory or inhibitory**									
**N-methyl-D-aspartate receptor (NMDAR) (1-3)**	21 (2 months-85 years)	1:4	Psychiatric syndrome, sleep disorders, seizures, amnesia followed by movement disorders, catatonia, autonomic instability, hypoventilation	Few cases with purely psychotic features; few with cryptogenic epilepsy	MRI: often normal or transient FLAIR or contrast enhanicng cortical or subcortical lesions. PET: relative frontal and temporal glucose hypermetabolism with occipital hypometabolism	Lymphocytosis in early stages (70%) and OCBs after (>50%); Abs usually present	EEG: frequent slow, disorganized activity (90%). Infrequent epileptic activity (20%). Rarely extreme delta brush pattern.	Ovarian teratoma in about 60%; post-HSV encephalitis (mainly children). Recently a few cases related to SARS-Cov2 infections have been reported.	~50% improve in 4 weeks with first line IT. 80% reach mRS 0–2. 12% relapsed within 2 years ~5% mortality.
**α-amino-3-hydroxy-5-methyl-4-isoxazolepropionic acid receptor (AMPAR) (4)**	55 (14-92)	2:1	LE with prominent seizures	Psychosis	Brain MRI: abnormal in 85% (usually bilateral temporal involvement)	Usually abnormal (75): lymphocytosis, OCBs; abs usually present	EEG: abnormali in 45%	Tumor in 70% cases (lung, thymoma, breast, ovary)	Most patients improve with IT; mortality related to underlying malignancy (15%)
**Gamma-aminobutyric acid A receptor (GABAAR) (5)**	40 (2 months-88 years)	1:1	LE with prominent seizures/status epilepticus	Psychiatric syndromes and catatonia; various presentation including SPS, opsoclonus, ataxia	Brain MRI: diffuse cortical and subcortical FLAIR signal abnormalities	Abnormal in up to 50% (lymphocytosis +/- OCBs); abs can be absent in the CSF	EEG: usually abnormal (80%) with epileptic activity and encephalopathy	Tumor in 15% cases (mostly thymoma)	Most patients improve with IT; mortality related to status epilepticus (20%)
**Gamma-aminobutyric acid B receptor (GABABR) (6)**	61 (16-67)	1.5:1	LE with prominent seizures	Ataxia, opsoclonus, status epilepticus	Brain MRI: abnormal in 70%	Common pleyocitosis (80%); rare OCBs. Abs usually present	EEG: usually abnormal (75%) with epileptic activity	Tumor in 50% (mainly lung)	Most patients improve with IT; mortality related to malignancy
**Metabotropic glutamate receptor 5 (mGluR5) (7)**	29 (6-75)	1.5:1	Encephalitis with psychiatric, cognitive, movement disorders, sleep dysfunction, and seizures	Ophelia syndrome	Brain MRI: abnormal in 50%	Lynphocitosis; abs presence unknown		Tumor (60%)(Hodgkin lymphoma, SCLC)	Response to IT
**Glycine receptor (GlyR) (8)**	50 (1-75)	1:1	Progressive encephalitis with rigidity and myoclonus or stiff person syndrome	LE, brainstem encephalitis; cryptogenic epilepsy	Brain MRI: mostly normal or non-specific; 5% temporal lobe inflammation. Spinal cord: lesions in 20%.	Pleocytosis in half of the cases, OCBs (20%); Abs can be absent in the CSF	EEG: 70% abnormal (mostly diffuse/focal slowing, 15% focal epileptic). EMG: continuous motor unit activity, spontaneous or stimulus-induced activity in 60%	Thymoma (15%)	Usually improve with IT.
**Antigens that modulate localization or function of potassium channels**									
**Leucine-rich glioma inactivated 1 (LGI1) (9-10)**	60 (30-80) but observed also in children	2:1	LE with or without FBDS and or generalized seizures	Cryptogenic epilepsy; neuromyotonia	MRI: medial temporal lobe hyperintensity (75%)	Usually normal, rare OCBs; abs can be absent	EEG: epileptiform activity in 30% of cases; focal slowing in 20%. Frequent hyponatremia (70%).	Tumor in 10% cases (mainly thymoma)	Despite recovery, cognitive deficits persist in many patients. One-third of patients relapse.
**Contactin-associated protein like 2 (CASPR2) (11)**	65 (25-77) but observed also in children	9:1	LE, MoS, NMT	Cerebellar ataxia, movement disorders, cryptogenic epilepsies, Guillain-Barre–like syndrome	MRI: medial temporal lobe hyperintensity (30%)	Usually normal (70%); rare OCB, pleocytosis and increased protein; abs can be absent	EEG: epileptiform activity in 40% of cases; focal slowing in 20%. Frequent hyponatremia (70%).	Tumor in 30% cases (mainly thymoma)	Response to immunotherapy. Relapse in 25% of cases.
**Dipeptidyl-peptidase-like protein-6 (DPPX) (12)**	53 (13-76)	1.5:1	Cognitive impairment, brainstem symptoms and diarrhea	Cerebellar ataxia, PERM	MRI: usually normal or non-specific	Pleocytosis, elevated proteins (30%); Abs usually present	EEG: 70% abnormal (mostly diffuse/focal slowing)	B cells tumor (10% cases)	Response to immunotherapy (70%)
**Antigen with likely cell-cell interaction functions but unclear overall role**									
**Ig-Like Domain-Containing Protein family member 5 (IgLON5) (13)**	64 (46-83)	1:1	NREM sleep disorder, abnormal movement and behaviours with obstructive sleep apnoea and stridor, gait instability and brainstem symptoms	Dementia, movement disorders (chorea); isolated dysphagia	MRI: usually normal or non-specific (80%)	Pleocytosis (30%), increased proteins (50%); Abs usually present		Tauopathy at neuropathology	Up to 50% respond to initial IT but a sustained response is rare.
**Neurexin3α (14)**	44 (23-57)	1:2	Prodromal fever, headache, or gastrointestinal symptoms, followed by confusion, seizures, and decreased level of consciousness		MRI: abnormal in 20% (mesial temporal involvement)	Pleocytosis in all cases			Elevated mortality (40%)
**Antigens normally considered to be associated with demyelinating disease and sometimes associated with encephalitic features**									
**AQP4 (15-16)**	32-41	5-10:1	NMOSD, LETM, ON	Area postrema syndrome, narcolepsy	Brain: frequent over time (85%); mainly medulla, hypothalamus and diencephalon. Spinal cord: usually LE lesions. Optic nerve: extensive, often involving chiasm and tracts.	Abnormal in up to 80% (pleocytosis, elevated protein); rare OCBs (10-15%).		Rare cancer association	Respond to IT but sequelae as well as relapses are frequent.
**MOG (17-18)**	37 (1-74)	1:1	NMOSD, LETM, ON, ADEM, TM	Encephalitis, brainstem encephalitis, seizures	Brain: abnormal in 75% (WM subcortical lesions +/- brainstem involvement) Spinal cord: abnormal in 50%; frequent conus medullaris involvement. Optic nerve: extensive, often bilateral lesions; frequent chiasmal involvement.	Abnormal in 60% (pleocytosis; rare OCBs).		Can be triggered by infections and vaccinations	Usually respond to corticosteroids (75%) Common relapses.

1. Dalmau J, Gleichman AJ, Hughes EG, Rossi JE, Peng X, Lai M, Dessain SK, Rosenfeld MR, Balice-Gordon R, Lynch DR. Anti-NMDA-receptor encephalitis: case series and analysis of the effects of antibodies. Lancet Neurol. 2008 Dec;7(12):1091-8. doi: 10.1016/S1474-4422(08)70224-2.

2. Titulaer MJ, McCracken L, Gabilondo I, et al. Treatment and prognostic factors for long-term outcome in patients with anti-NMDA receptor encephalitis: an observational cohort study. Lancet Neurol 2013;12:157–65. doi: 10.1016/S1474-4422(12)70310-1

3. Zandifar A, Badrfam R. COVID-19 and anti-N-methyl-d-aspartate receptor (anti-NMDAR) encephalitis: Are we facing an increase in the prevalence of autoimmune encephalitis? J Med Virol. 2021 Apr;93(4):1913-1914. doi: 10.1002/jmv.26745.

4. Laurido-Soto O, Brier MR, Simon LE, et al. Patient characteristics and outcome associations in AMPA receptor encephalitis. J Neurol 2019;266:450–60. doi: 10.1007/s00415-018-9153-8.

5. Spatola M, Petit-Pedrol M, Simabukuro MM, Castro FJ, et al. Investigations in GABAA receptor antibody-associated encephalitis. Neurology 2017;88:1012–20.

6. Lancaster E, Lai M, Peng X, et al. Antibodies to the GABA(B) receptor in limbic encephalitis with seizures: case series and characterisation of the antigen. Lancet Neurol 2010;9:67–76. doi: 10.1016/S1474-4422(09)70324-2.

7. Spatola M, Sabater L, Planagumà J, et al. Encephalitis with mGluR5 antibodies: Symptoms and antibody effects. Neurology. 2018;90(22):e1964-e1972. doi: 10.1212/WNL.0000000000005614.

8. Carvajal-González A, Leite MI, Waters P, et al. Glycine receptor antibodies in perm and related syndromes: characteristics, clinical features and outcomes. Brain 2014;137:2178–92. doi: 10.1093/brain/awu142.

9. Irani SR, Michell AW, Lang B, Pettingill P, Waters P, Johnson MR, Schott JM, Armstrong RJ, S Zagami A, Bleasel A, Somerville ER, Smith SM, Vincent A. Faciobrachial dystonic seizures precede Lgi1 antibody limbic encephalitis. Ann Neurol. 2011 May;69(5):892-900. doi: 10.1002/ana.22307.

10. Ariño H, Armangué T, Petit-Pedrol M, et al. Anti-LGI1-associated cognitive impairment: presentation and long-term outcome. Neurology 2016;87:759–65. doi: 10.1212/WNL.0000000000003009.

11. Irani SR, Pettingill P, Kleopa KA, et al. Morvan syndrome: clinical and serological observations in 29 cases. Ann Neurol 2012;72:241–55. doi: 10.1002/ana.23577.

12. Tobin WO, Lennon VA, Komorowski L, et al. Dppx potassium channel antibody: frequency, clinical accompaniments, and outcomes in 20 patients. Neurology 2014;83:1797–803. doi: 10.1212/WNL.0000000000000991.

13. Gaig C, Graus F, Compta Y, et al. Clinical manifestations of the anti-IgLON5 disease. Neurology 2017;88:1736–43. doi: 10.1212/WNL.0000000000003887.

14. Gresa-Arribas N, Planagumà J, Petit-Pedrol M, et al. Human neurexin-3α antibodies associate with encephalitis and alter synapse development. Neurology 2016;86:2235–42. doi: 10.1212/WNL.0000000000002775.

15. Wingerchuk DM, Banwell B, Bennett JL, Cabre P, Carroll W, Chitnis T, de Seze J, Fujihara K, Greenberg B, Jacob A, Jarius S, Lana-Peixoto M, Levy M, Simon JH, Tenembaum S, Traboulsee AL, Waters P, Wellik KE, Weinshenker BG; International Panel for NMO Diagnosis. International consensus diagnostic criteria for neuromyelitis optica spectrum disorders. Neurology. 2015 Jul 14;85(2):177-89. doi: 10.1212/WNL.0000000000001729.

16. Wingerchuk DM, Hogancamp WF, O’Brien PC, Weinshenker BG. The clinical course of neuromyelitis optica (Devic’s syndrome). Neurology. 1999 Sep 22;53(5):1107-14. doi: 10.1212/wnl.53.5.1107.

17. Juryńczyk M, Jacob A, Fujihara K, et al. Myelin oligodendrocyte glycoprotein (MOG) antibody-associated disease: practical considerations Practical Neurology 2019;19:187-195.

18. Hamid SHM, Whittam D, Saviour M, et al. Seizures and encephalitis in myelin oligodendrocyte glycoprotein IgG disease vs aquaporin 4 IgG disease. JAMA Neurol 2018;75:65–71. doi: 10.1001/jamaneurol.2017.3196 pmid: http://www.ncbi.nlm.nih.gov/pubmed/29131884.

NMDAR-Ab encephalitis (NMDARE) is one of the most common forms of autoimmune encephalitis, with an estimated incidence of 1.5 per million person-year (149). An American cohort found a prevalence of 0.6/100000 people, with a generally higher prevalence of autoimmune encephalitis in African Americans compared to Caucasian subjects (150). No data of the epidemiology of autoimmune encephalitis are available for Africa, and only very limited cases have been reported so far, outlining possible difficulties in achieving the diagnosis (151). NMDAR-Ab encephalitis can affect both children and adults, and although clinical presentations can vary with age (152), it is associated in most cases with a predictable set of symptoms. The multistage characteristic clinical syndrome is usually preceded by prodromic manifestations such as fever, headache, or viral-like illness. This is then followed, within one to three weeks, by psychiatric manifestations, sleep disturbances, memory impairment, seizures, language dysfunction, and in many cases with catatonia, dyskinesias, autonomic instability, decreased level of consciousness, and central hypoventilation.

#### 3.1.1 Triggers and Neuroimmunology

The disease is related to the presence of antibodies directed against the NR1 subunit of the NMDA receptor. These antibodies, mainly IgG1, have been shown to cause NMDAR internalization and reduced expression (similar to that in myasthenia gravis, see below) and have been demonstrated to be pathogenic by *in vitro* (153) and *in vivo* studies (154, 155).

The initial reports of NMDARE described a few cases of young women with psychiatric abnormalities, movement disorders and central hypoventilation in association with ovarian teratoma (156, 157). A subsequent study showed that about 50% of female patients with NMDAR-Ab encephalitis over 18 years bear uni- or bi-lateral ovarian teratomas (158). Compared to teratomas of patients without encephalitis, the teratomas of patients with NMDAR antibodies more often contain lymphoid structures characterized by aggregates of B and T cells, plasma cells and mature dendritic cells (159–163), and abnormal neuroglial tissue (161, 164), that expresses the NMDAR antibody subunit target NR1 (159). Moreover, the infiltrating B cells were shown to produce NMDAR antibodies *in vitro* (159) supporting a primary role of these tumour resident immune cells in the generation of the antibodies and explaining the patients’ partial clinical improvement after tumour removal (158).

Besides ovarian teratoma, herpes simplex viral encephalitis (HSVE) is now considered a well-established possible trigger of NMDARE (165). A prospective cohort study showed that 27% of patients with HSVE developed symptoms of AE within 3 months. Patients who developed detectable neuronal antibodies within 3 weeks from onset had higher risk of autoimmune encephalitis. Clinical features and outcome were age dependent, with children 4 years old or younger more likely to develop choreoathetosis, decreased level of consciousness and frequent seizures or infantile spasms, responding less to immunotherapies compared to older children and adults who developed predominant change of behaviour and psychiatric symptoms sometimes accompanied by seizures. Overall, the outcome of post-HSV encephalitis, particularly in younger children, was worse than that of patients with classical NMDAR-antibody encephalitis, although the reason is unclear (165). Several mechanisms, including blood-barrier disruption with increased inflammation and complement deposition, T-cell mediated cytotoxicity, the presence of viral related damage, have been implicated but need confirmation (165).

Similarly, the mechanism underlying post-HSV encephalitis must be clarified. It is possible that molecular mimicry between NMDAR and HSV proteins play a major role. However, the frequent presence of other NSA-Ab (165–168) suggests that other mechanisms might be more likely, such as a secondary release of antigenic proteins from neuronal injury or host inflammatory responses specific to HSV infection. Other infections, including varicella zoster (169), Japanese encephalitis (170), HIV (171) and recently Covid-19 (172–174), have been reported as triggers of NMDAR antibodies as well as other AE, suggesting a model where a viral-induced brain destructive inflammatory process causes the release of neuronal surface proteins and receptors which become secondary targeted antigens of the virus-triggered immune response. The host HLA genetic background could be relevant to whether the HSVE patient develops AE or not.

The diagnosis of NMDARE is confirmed by the detection of IgG antibodies to NR1 in the serum or CSF. The latter is considered highly sensitive and specific for NMDARE (175). CSF analysis can show lymphocytic pleocytosis or oligoclonal bands, although it can show normal finding at onset (158, 175). EEG often shows diffuse slow and disorganized activity, and some epileptic discharges (175). A unique EEG pattern, defined extreme delta waves, can be found in a subgroup of patients and it is considered highly suggestive the diagnosis (176). Brain MRI can be normal or show multiple abnormalities in cortical and subcortical regions in FLAIR with possible contrast enhancement (175). It must be noted that access to brain MRI and antibody testing might be limited in some African situations, hampering the achievement of a correct diagnosis (151, 177).

Since the prognosis of NMDARE is largely time dependent (158), early diagnosis and treatment are pivotal to ensure a good outcome. For this reason, in 2016, a consensus of experts established a set of clinical criteria to help clinician to achieve a diagnosis of probable NMDARE, even without the confirmatory detection of the antibodies which could not be always easily or timely available, although it remains fundamental for the definite diagnosis (178). In a retrospective paediatric cohort, these criteria shown 90% sensitivity and 96% specificity for the diagnosis of probable NMDARE, after a median of 2 weeks from onset (179). Another study, including both children and adults, showed a sensitivity of 49% and a specificity of 98% for the diagnosis of probable NMDARE. Also in this case, the sensitivity increased over time from 16% in the first 2 weeks to reach 87% between 31 and 90 days after onset (180). Differential diagnosis includes mainly viral encephalitis, malignant catatonia, neuroleptic malignant syndrome, and primary psychiatric disorders. Clinical features distinguishing HSVE from non-viral and post-HSV NMDARE are shown in **Table 3**.

**Table 3:** Clinical features and differential diagnosis between NMDARE, HSVE and relapse and post-HSVE NMDARE.

Clinical features	NMDARE	HSV encephalitis	HSVE relapse	Post-HSVE NMDARE
Prodromes Main syndrome	Headache, fever, diarrhea, flu-like syndrome		Previous HSVE (usually within 3 weeks)	Previous HSVE (within 2-16 weeks)
	Psychiatric syndrome, sleep disorders, seizures, amnesia followed by movement disorders, catatonia, autonomic instability, hypoventilation	Seizures, headache, confusion, fever, personality changes/psychiatric symptoms	Fever, seizure, altered level of consciousness.	Frequent movement disorders, altered level of consciousness (particularly in children); more frequently seizures and psychiatric disorders in adults.
Brain MRI	Often normal or transient FLAIR or contrast enhancing cortical or subcortical lesions.	Frequent (90%) uni- or bi-lateral temporo-mesial T2/FLAIR hyperintensities	Frequent uni- or bilateral lesion; frequent new lesions with edema, hemorrhage, and necrosis in the inferomedial temporal lobe.	Contrast enhancement comparable to that found during the viral encephalitis.
CSF	Lymphocytosis in early stages (70%) and OCBs after (>50%)	Pleocytosis, increased protein; frequent red blood cells.	Pleocytosis, increased protein; frequent red blood cells.	Pleocytosis, increased protein.
EEG	Frequent slow, disorganized activity (90%). Infrequent epileptic activity (20%). Rarely extreme delta brush pattern.	Abnormal in 80% (paroxysmal spike and sharp waves). Temporal triphasic waves and PLEDs.	Usually altered; frequent worsening bilateral abnormality with slow wave activity and recurrent periodic complexes.	Can be slow, normal or show epileptic activity
Diagnostic tests	NMDAR antibodies in CSF +/- in serum	HSV PCR in CSF. Possible false negative (early stages; children)	HSV PCR in CSF	NMDAR antibodies in CSF; HSV PCR usually negative
Outcome	~50% improve in 4 weeks with first line IT; 80% reach mRS 0–2; 12% relapsed within 2 years ~5% mortality.	Frequent neurological sequelae, high mortality and morbidity	Frequent neurological sequelae, high mortality and morbidity	Neurological sequelae more frequent and more severe than classical NMDAR encephalitis

Early recognition of post-HSVE NMDARE is relevant to ensure timely initiation of immunosuppressive treatment and better outcomes. This diagnosis should be suspected in patients, and particularly children, with relapsing symptoms post HSVE and confirmed by NMDAR-antibody detection in the CSF, since serum antibodies can occur post HSVE also in patients without encephalitis (165). No brain MRI or CSF features during the acute HSV infection appear to predict the onset of post-HSVE NMDARE. Brain MRI studies at onset of autoimmune encephalitis showed that 82% had contrast enhancement comparable to that found during the viral encephalitis, similarly to findings observed in patients who did not develop post-HSVE encephalitis. However, patients who developed autoimmune encephalitis were more likely to have necrosis with cystic lesions in MRIs obtained at follow-ups (165). At onset of the post-HSVE NMDAR encephalitis, CSF HSV1–2 PCR is generally negative, showing mild pleocytosis and increased protein levels (165, 167). The limited cases with concomitant CSF detection of HSV by PCR and NMDAR antibodies had clinical phenotypes compatible with the autoimmune disease (181).

#### 3.1.2 Treatment

NMDARE treatment is based on immunosuppression and tumour removal in paraneoplastic cases. Immunotherapy involves an escalation from first-line therapies (steroids, intravenous immunoglobulin, or plasma exchange) towards second-line treatments (rituximab or cyclophosphamide) in non-responders. About 50% of 472 patients who underwent first-line treatment or tumour removal showed an improvement with 4 weeks. Among those who did not improve 57% received a second-line treatment which resulted in a better outcome compared to those who did not receive second-line. Around 10% of patients are refractory to second-line therapies (158). In these cases, bortezomib or tocilizumab have been suggested as third-line therapies (182, 183). Overall, about 75% of patients experience only mild long-term deficits or recover completely, but the remaining 25% have severe sequelae, and mortality due to intensive care complications can be up to 7% (158, 184, 185). Relapses occur in 12% of patients and are more frequent in non-paraneoplastic cases and in patients who did not receive a second-line treatment (158). Again, it must be underlined that access to plasmapheresis and immunoglobulin might be difficult in some African regions (151). Moreover, ICU might not be always available and when it is, mortality risk in ICU is high, mainly in relation to sepsis and tracheostomy requirements (177, 186).

In patients with suspected post-HSVE NMDARE, antiviral therapy should be started, until a relapse of HSV encephalitis is excluded. Once the diagnosis is established, first- and/or second-line immunotherapy should be promptly started. Immune treatment has not been associated with HSV encephalitis relapse (181). It is unclear if early steroid treatment during HSV could decrease the risk of secondary autoimmunity, but a clinical trial is under way (187). Therefore, to date, early steroid and acyclovir combination therapy remains experimental (188).

## 4 A Recognised Autoantibody-Mediated Disorder Without Known Relationship to Infection

### 4.1 Myasthenia Gravis

Myasthenia gravis (MG) is the archetypal autoantibody-mediated disease. It is relatively rare with an estimated annual incidence of 8–10 cases per million person-years (189). It is characterized by fatigable muscle weakness due to loss of acetylcholine receptors (AChRs) at the neuromuscular junction (190). AChR loss is due either to antibodies directly binding the receptor (AChR-Abs) or to antibodies inhibiting the function of muscle specific kinase (MuSK-Abs) which regulates AChR numbers and density. A proportion of patients have thymic hyperplasia or a thymic tumour but otherwise the aetiology is unknown. Other neuromuscular junction disorders are described in **Table 4**.

**Table 4:** Disorders of neuromuscular transmission and differential diagnoses.

	Main clinical features	Basic treatments
**Myasthenia gravis**		
AChR antibodies Younger females (<50y) and older males (>50y).	Generalised or more localised weakness and fatigue. Increases on repetitive activity. Thymic hyperplasia; must look for thymoma but many older patients have no thymoma or hyperplasia.	Anti-cholinesterase. Steroids and azathioprine. Plasma exchange if available
MuSK antibodies	Often more bulbar and respiratory than generalised weakness.	Anti-cholinesterases can be detrimental. Plasma exchange very effective, steroids and azathioprine not always adequate.
**Lambert-Eaton Myasthenic Syndrome**		
VGCC antibodies	Weakness that decreases with brief tonic activity. Strongly associated with small cell lung cancer and smoking history, but some patients have no tumour and a purely autoimmune disease.	3,4-di-amino-pyridine helpful but difficult to acquire. Steroids and azathioprine as for MG.
	Often neuromuscular junction effects with weakness in ocular and respiratory muscles.	As per local guidelines
**Important Differential Diagnoses** **a. Venoms and Neurotoxins eg. snake bite, botulism, tetanus**
**b. Congenital Myasthenic Syndromes**	Inheritance mostly autosomal recessive but autosomal dominant in a few. Diverse neuromuscular junction gene mutations in pre and postsynaptic proteins particularly choline acetylase, Collagen Q, AChR, MuSK, DOK7 and others. Not always clinically evident in early life and older onset genetic disorders can be misdiagnosed as autoimmune MG. If suspected, refer to Rodriguez Cruz et al., 2018 for details	Treatment is symptomatic and mutation analysis is helpful in defining treatments for different forms which can respond adversely to the incorrect therapy, eg. anticholinesterase drugs make some conditions worse.

MG has been widely reported around the world. In South Africa, the incidence figures, age and gender distributions were similar to reports from Europe and North America (191), with a bimodal pattern: mainly females peaking at 30 years at onset and a higher peak at 70–80 years of age with predominantly males. The apparent “increase” in the incidence, compared with a decade previously, was likely due to the greater availability of the AChR-ab testing in addition to better access to specialist healthcare (191, 192). The standardized incidence rate for childhood AChR-Ab MG in a South African study was higher than a report from England (193). Childhood MG is also common in East Asia (191, 194). Most data available in other Sub-Saharan countries comes from small series and case reports (195–197), and MG may be unrecognised and untreated in large parts of Africa.

Clinically MG is characterized by fatigable weakness of ocular, bulbar, and proximal limbs muscle, which is often worse at the end of the day and in milder cases may improve with brief rest periods. Classically, around 15% of MG patients have pure ocular symptoms (double vision and ptosis), and many of the 85% with generalized MG may initially present with ocular symptoms. Fatigable bulbar symptoms include chewing fatigue, swallowing and speech difficulties. Chest muscle and diaphragm involvement can result in insidious, asymptomatic type II respiratory failure with early morning headaches and cor pulmonale. The selective involvement of triceps muscle weakness was described in a small group of African Americans with MG (198) and has also been seen in those with African genetic ancestry of whom 15 of 96 (16%) also had distal finger extensor weakness (Heckmann, unpublished observations). In addition, in South Africa MG patients with African genetic ancestry, both adults with juvenile onset disease and children, are more prone to develop treatment resistant ophthalmoplegia and ptosis (199, 200). Unbiased genome-wide sequencing studies in AChR-ab positive MG patients, with and without the ophthalmoplegic sub-phenotype of MG, have shown association with several muscle-expressed genes known to be involved in muscle atrophy signalling and myosin II function (201, 202). Gene expression studies in the orbital muscles of affected MG patients vs controls, also showed aberrant regulation of muscle atrophy and mitochondrial pathways (203). The importance of these findings for the treating physician is that in MG-induced ocular muscle paralysis, early intervention with immune treatment with the aim of minimizing the duration of ocular muscle paralysis, has shown the best treatment responses (204).

Autoantibodies against the muscle AChR are predominantly IgG1 and IgG3 subclasses and lead to loss of AChRs by two main mechanisms; by complement activation and by cross-linking and internalization of AChRs (205). Patients can improve rapidly when the antibodies are reduced in concentration by using plasma exchange and steroids. MuSK autoantibodies, by contrast, are predominantly of the IgG4 subtype. They are found in a proportion of patients without AChR antibodies and have a relatively high incidence of bulbar involvement and often respond poorly to immunotherapies (206). In Europe, there is a north-south gradient with MuSK-Ab MG being more common in Mediterranean countries. In patients with African genetic ancestry, either indigenous African (black) or mixed African genetic ancestry, studies from North America (211, A Vincent unpublished) and South Africa (207) have reported a higher proportion of AChR-Ab negative patients with MuSK antibodies. Future studies within the African continent are needed to increase our knowledge of the epidemiology and distribution of MG autoantibodies.

#### 4.1.1 Management and Treatment of MG in Sub-Saharan Africa

MG diagnosis is primarily clinically based although antibody testing can be helpful to confirm the diagnosis and for subgroup classification (208). However, these serological tests are not widely available in many sub-Saharan African countries, and shipping the tests abroad is expensive so most physicians must rely on recognition of the clinical features, neurophysiological studies if available, and reversibility of the symptoms by treatments, to help establish the diagnosis.

The main treatments are cholinesterase inhibitors, that temporarily reverse symptoms, and steroids that reduce the antibody levels. The steroid-sparing agent azathioprine is generally available, as it is included in the WHO list of essential medicines (209), while mycophenolate mofetil, methotrexate, tacrolimus and other immunosuppressive agents can be difficult to find. However, methotrexate as a steroid-sparing agent for newly diagnosed MG, was found to be as safe and effective as azathioprine and is 10-fold cheaper than azathioprine (210). Importantly, methotrexate (and mycophenolate mofetil) must be avoided in potentially child-bearing women, but it is useful in children and older people with MG. The availability of anti-CD20 monoclonal antibody rituximab for patients with refractory AChR-antibody positive MG and MuSK-MG is limited due to its high costs. However, the use of single low doses of rituximab has proven very effective for 6-9 months or longer, in a cohort of refractory cases from South Africa, including myasthenic patients living with HIV (Heckmann, unpublished). It is important to be aware that with limited critical care capacity in low and lower middle-income African countries (211), routine follow-ups and close monitoring of immunosuppressive therapies are crucial to minimize the risk of myasthenic exacerbations. Certain antibiotics may trigger MG crises and should be avoided including tetracyclines, fluoroquinolones (and quinine), and aminoglycosides (https://www.myastheniagravis.org/mg-and-drug-interactions/). Artesunate has been used to successfully treat malaria in an MG patient (212).

MG remains a rare disease and there are numerous challenges to clinical trials, such as poor recruitment of participants (213). Currently, <2% of clinical trials worldwide take place in Africa, mainly in Egypt and South Africa (214). Establishing well-characterised cohorts and registries of MG patients in sub-Saharan Africa could help patients benefit from the development of new therapies, and also advance clinical trials globally for MG.

Other challenges in managing patients with MG in sub-Saharan Africa include concomitant infectious diseases such tuberculosis, Human Immunodeficiency Virus (HIV) infection and hepatitis B/C coinfection, which are prevalent in some African countries. Screening for these infections prior to immunomodulatory treatment are essential and prophylactic treatment for tuberculosis, such as isoniazid with pyridoxine supplementation for 6-9 months, should be considered in MG patients who have evidence of scarring on their chest radiographs when immunosuppression is started (215). The risk of reactivation of latent tuberculosis is highest in the first year of starting immunotherapies, and particularly with higher doses of steroids (216). Overall, the therapeutic approach to MG patients with HIV infections should be similar to those who are uninfected. Worsening of MG within 6 months of starting antiretroviral treatment can be seen as an effect of immune recovery (215). Monitoring MG patients with HIV infection receiving immune therapies, should include 6-monthly HIV viral load estimation to ensure effectiveness of antiretroviral therapy. As with most chronic diseases, monitoring of the patient’s disease is useful to direct clinical decision making. The MG-activities of daily living (MG-ADL) is a simple, validated questionnaire which could be used in African settings (217).

## 5 Conclusion

Unravelling the interplay between viral infections, neurological autoimmunity and genetics is work in progress. Viruses need to access the host nucleus to replicate and cause disease. Answers regarding the role of host mutations in RANBP2, a nuclear pore protein, involved in the pathogenesis of recurrent ANE1 makes this condition a very good model for understanding the links between genetics, viral infections and neuroinflammation. Our understanding of the role of viral mutations in the enhanced fusion with CNS target cells and pathogenesis of persistence of MeV in MIBE and SSPE is important for development of future therapies for these devastating MeV CNS complications. There are lots of other unanswered questions regarding recurrence in Guillain-Barre syndrome, the “cytokine storm” target cells and pathogenesis on ANE/NE1, the causal link between Onchocerciasis and associated neurological syndromes, etc. Lessons learnt from well-studied models like myasthenia gravis and autoimmune encephalitis are important in shedding light on the basic immune principles and therapeutic possibilities for both CNS and PNS post-infectious autoimmune diseases.

There are many gaps in knowledge regarding post-infectious autoimmunity in the nervous system. The African continent faces serious challenges in tackling not only the endemic, epidemic and pandemic infections, but the immunological conditions that are sequelae of these infections. Examples of challenges are lack of data, infrastructure, tools, health and scientific research personnel, political stability, etc. Pandemic collision is a real threat that could result in catastrophic human life and economic losses.

There are also things that are relatively easy to do, the proverbial “low-hanging fruit”. There is much that Africa can easily achieve with the current limited resources. Some answers are readily available, like the simple evidence that vaccination works. Measles, which still ravages many parts of the continent is preventable (218). The best way to manage the neurological complications of measles is to vaccinate young infants, achieve high vaccine coverage and to promote herd immunity (219). Evidence is also mounting regarding the efficacy and effectiveness of vaccines in preventing Covid-19 morbidity and mortality. Resources must be pooled to bolster vaccine initiatives and expedite roll-out. Education and public campaigns about the importance of vaccination and creation of an atmosphere and infrastructure that enable it are essential. Vaccine hesitancy must be addressed. Political will is required from governments across Africa with continental and intercontinental collaboration. International pressure needs to be mounted to discourage western governments from hoarding resources like vaccines.

There are other silver linings that need to be pursued. A lot of lessons have been learnt in the past when dealing with previous endemics/epidemics like, HIV, malaria, and Ebola. The know-how and health infrastructures built over time to address these scourges in many African countries, must be readapted and used as “tram-tracks” for new programmes to deal with new and emerging pandemics. Data gaps must be addressed, integrated disease surveillance increased, and reporting escalated through multidisciplinary, national, and international collaboration. Investing in the youth of the continent, training young future health scientists armed with modern skills and tools to face future challenges will go a long way.

## Author Contributions

JW: planning article structure and manuscript review and editing. AK-M: planning article structure, subsection author, and manuscript draft and review. AV: planning article structure, abstract review, subsection author, and manuscript draft/editing. HW: planning article structure, subsection author, and manuscript draft and review. KB: planning article structure, subsection author, and manuscript review. BE: planning article structure, subsection author, manuscript draft, and review and editing. AN: corresponding author, planning article structure, drafting abstract, subsection author, and manuscript draft and editing. JH: co-author of a section and manuscript review and editorial input overall. PC: planning article structure, subsection co-author, and manuscript review. MG: planning article structure, subsection author, and manuscript draft and review.

## Conflict of Interest

The authors declare that the research was conducted in the absence of any commercial or financial relationships that could be construed as a potential conflict of interest.

## Publisher’s Note

All claims expressed in this article are solely those of the authors and do not necessarily represent those of their affiliated organizations, or those of the publisher, the editors and the reviewers. Any product that may be evaluated in this article, or claim that may be made by its manufacturer, is not guaranteed or endorsed by the publisher.
